# Accumulation patterns of tobacco root allelopathicals across different cropping durations and their correlation with continuous cropping challenges

**DOI:** 10.3389/fpls.2024.1326942

**Published:** 2024-03-12

**Authors:** Fangfang Zhou, Yihong Pan, Xiaolong Zhang, Guobing Deng, Xiaoting Li, Yubin Xiong, Li Tang

**Affiliations:** ^1^ College of Plant Protection, Yunnan Agricultural University, Kunming, China; ^2^ College of Materials and Chemical Engineering, Southwest Forestry University, Kunming, China; ^3^ Yunnan Wooja Bio-tech Co.Ltd., Kunming, China

**Keywords:** continuous cropping obstacles, allelopathicals, tobacco black shank, beneficial bacteria, rhizosphere

## Abstract

**Introduction:**

Continuous cropping challenges have gradually emerged as pivotal factors limiting the sustainable development of agricultural production. Allelopathicals are considered to be the primary obstacles. However, there is limited information on allelopathic accumulation across various continuous cropping years and its correlation with the associated challenges.

**Methods:**

Tobacco was subjected to varying planting durations: 1 year (CR), 5 years (CC5), 10 years (CC10), and 15 years (CC15).

**Results:**

Our findings unveiled discernible disparities in tobacco growth patterns across diverse continuous cropping periods. Notably, the most pronounced challenges were observed in the CC5 category, characterized by yield reduction, tobacco black shank outbreaks, and a decline in beneficial flora. Conversely, CC15 exhibited a substantial reduction in challenges as the continuous cropping persisted with no significant differences when compared to CR. Within the tobacco rhizosphere, we identified 14 distinct allelopathic compounds, with 10 of these compounds displaying noteworthy variations among the four treatments. Redundancy analysis (RDA) revealed that eight allelopathic compounds exhibited autotoxic effects on tobacco growth, with MA, heptadecanoic acid, and VA ranking as the most potent inhibitors. Interaction network highlighted the pivotal roles of VA and EA in promoting pathogen proliferation and impeding the enrichment of 13 beneficial bacterial genera. Furthermore, a structural equation model elucidated that MA and EA primarily exert direct toxic effects on tobacco, whereas VA fosters pathogen proliferation, inhibits the enrichment of beneficial bacteria, and synergistically exacerbates the challenges associated with continuous cropping alongside EA.

**Discussion:**

These findings suggested discernible disparities in tobacco growth patterns across the various continuous cropping periods. The most pronounced challenges were observed in CC5, whereas CC15 exhibited a substantial reduction in challenges as continuous cropping persisted. VA may play a pivotal role in this phenomenon by interacting with pathogens, beneficial bacterial genera, and EA.

## 1 Introduction

There is an urgent need to alleviate continuous cropping obstacles worldwide (Li et al., 2019). Long-term continuous cropping leads to crop yield reduction, soil-borne disease outbreaks, and soil degradation (Qin et al., 2017), including nutrient imbalances, soil acidification, and disruptions in microbial community structures (Xi et al., 2019), which threaten the sustainable development of agricultural production (Liu et al., 2018).

It is widely considered that soil nutrient imbalances, disruption of microbial communities, and allelopathic autotoxicity are the most important factors for continuous cropping challenges (Deng et al., 2017; Bai et al., 2019; Ao et al., 2022). Allelochemicals, a form of root exudates, play direct (Bortolo et al., 2018) or indirect (Ren et al., 2017) roles in consecutive monoculture problems. These allelopathic compounds, mainly plant secondary metabolites such as terpenes (Zhao et al., 2018), phenolic acids (Rial et al., 2014), and saponins (Li et al., 2020), contribute to autotoxicity. Numerous allelopathicals have been reported linked to continuous cropping challenges, encompassing vanillic acid, vanillin, cinnamic acid, ferulic acid, saponin, and benzoic acid (Li et al., 2012; Ni et al., 2012; Yeasmin et al., 2014; Deng et al., 2023). In cucumber, cinnamic acid is a primary contributor to the consecutive monoculture problem (Xiao et al., 2020). Autotoxic ginsenosides alter the soil fungal community and lead to replanting failure in Sanqi (Li et al., 2020). Although phytotoxic metabolites released from *Rehmannia gulutinosa* are the main cause of replanting, the specific key allelochemicals remain unknown (Zhang et al., 2019). Eight allelochemicals have been detected in *A. lancea* roots, which exhibit significant autotoxicity during germination and seedling growth (Wang et al., 2023). Tobacco is a globally significant model plant and a major cash crop that contains abundant secondary metabolites, making it more prone to allelopathic activity (Deng et al., 2017). For example, exudates from tobacco rhizosphere soil after 12 consecutive years of continuous cropping negatively affects lettuce and tobacco seedlings, demonstrating strong allelopathy (Wu et al., 2015). Furthermore, di-*n*-butyl phthalate and disobutyl phthalate have been confirmed as autotoxins in tobacco rhizosphere (Deng et al., 2017). These results show that long-term continuous cropping results in the secretion of substances responsible for continuous cropping obstacles. Therefore, it is imperative to explore allelopathic accumulation patterns and the underlying mechanisms causing continuous cropping challenges.

Wu et al. (2015) confirmed that allelopathicals in lily rhizosphere soil increased with continuous cropping years, resulting in the inhibition of beneficial bacterial growth and adverse effects on crop development (Kumar et al., 2017). In the cucumber rhizosphere, vanillic acid, a primary allelopathic compound, modulates the composition and diversity of *Trichoderma* and *Fusarium* spp., thereby affecting plant growth (Zhou and Wu, 2018). Interactions between allelopathicals and pathogenic microorganisms can exacerbate plant diseases. The addition of 0.1 mmol/L benzoic acid externally promoted mycelial growth, sporulation ability, and conidial germination of *Fusarium solani*, causing peanut root rot (Liu et al., 2016). Gallic acid, p-hydroxybenzoic acid, and ortho-hydroxybenzoic acid can trigger soil-borne disease outbreaks, such as *Fusarium wilt* and *Verticillium wilt*, by stimulating pathogen spore germination (Zhang et al., 2012). These have aggravated continuous cropping obstacles. Meanwhile, interactions between allelopathicals and beneficial bacteria may be linked to continuous cropping obstacles. For example, autotoxins can simplify microbial structure and reduce the abundance of dominant microorganisms (Chen et al., 2018).

The above studies confirmed that allelochemicals showed an enrichment trend under continuous cropping and interacted with pathogens, leading to the occurrence of diseases (Chen et al., 2023). Conversely, certain crops exhibit the ability to enrich beneficial microorganisms over prolonged continuous cultivation, creating a soil legacy that protects subsequent crops from pathogenic bacteri (Jos and Raaijmakers, 2016; Bakker et al., 2018). For instance, in the case of continuous wheat cropping, the occurrence of wheat root rot has diminished over several years, attributed to the recruitment of antagonistic fluorescent *Pseudomonas* spp. These microorganisms release the antifungal compound 2,4-diacetylphlologlucino (David et al., 2002). Root exudates, including allelopathic substances, play a significant role in forming a soil legacy that enhances crop disease resistance (Yuan et al., 2018).However, the relationship among allelopathic substances, disease occurrence, and accumulation patterns across different continuous cropping years, and their connection to long-term continuous cropping challenges remain unclear. In this study, we identified 14 allelochemicals in tobacco rhizosphere using GC-MS. We examined how the accumulation of these allelochemicals changed with the duration of tobacco continuous cropping. Additionally, we analyzed the interplay between these allelochemicals, tobacco growth, major soil-borne diseases, and antagonistic bacteria. The goal was to elucidate the key allelopathicals throughout different durations of continuous cropping and their potential mechanisms in causing continuous cropping obstacles.

## 2 Materials and methods

### 2.1 Study area description and soil sampling collection

The experimental plots were located in Baishui Town, Luxi County, Yunnan Province, China (103.8727°E, 24.6657°N). Luxi County is an important tobacco-producing area in Yunnan Province, where approximately 10,000 hm^2^ of flue-cured tobacco are cultivated annually. Baishui Town, located in the northeastern part of Luxi County, experiences a subtropical monsoon climate characterized by an annual average temperature of 15°C, annual average precipitation of 1031.7mm, annual average frost-free period lasting 238 days, and an average of 1962.9 h of sunshine annually. These conditions make it well suited for high-quality tobacco leaf production. Four experimental plots were established, each with size of 667 m^2^, all located in the same area and managed by the same farmer. The planting scenarios included tobacco planted in the first year (no previous tobacco history, CR), continuous tobacco planting for 5 years (since 2016, CC5), continuous tobacco planting for 10 years (since 2011, CC10), and continuous tobacco planting for 15 years (since 2006, CC15). Tobacco transplantation should occur annually between April 15th and May 10^th^, with tobacco harvesting being completed before September. All plots used the Yunyan 87, a cultivar known for its moderate resistant to black shank disease (Peng et al., 2015). The natural soil in the region is classified as red soil, according to the Chinese soil classification. The planting density was set at 12,000 tobacco plants per ha. For fertilizer application, a uniform dosage of 105 kg/hm^2^ of pure nitrogen to the study area. A compound fertilizer at a rate of 75 kg/hm^2^ (N:P_2_O_5_:K_2_O = 12:8:25) was used during transplanting. For the top dressing, 30 kg/hm^2^ of compound fertilizer (N:P_2_O_5_:K_2_O = 12:8:25) was applied, followed by 150 kg/hm^2^ of potassium sulfate (K_2_O ≥ 52%) for the second top dressing. The other management practices remained consistent across all plots.

In September 2020, we collected tobacco rhizosphere soil from the four experimental plots. Each soil sample consisted of five-point samples from within each plot, collected in an “S” shape pattern. After carefully excavating the entire tobacco root system and gently removing any large soil clumps, we combined the soil immediately surrounding the root surface from these five locations into a composite sample for each plot. This process was repeated six times for each treatment. To maintain sample quality, we promptly transported the soil to the laboratory in an icebox, passed it through a 2-mm sieve, and then divided it into three portions, all of which were stored at -80°C. One portion was dedicated to DNA extraction, and qPCR was used for quantitative detection of tobacco black shank pathogens. The remaining portion was used to analyze the presence of allelopathicals in the soil.

### 2.2 Total soil DNA extraction, PCR amplification, and high-throughput sequencing

Soil DNA was extracted using the DNeasy Power Soil Kit (Qiagen, Hilden, Germany). The 16S rRNA genes were amplified using specific primers, namely 338F (5’-ACTCCTACGGGAGMHAMHA-3’) and 806R (5’-GGACTACHVGGGTWTCTAAT-3’), along with ITS1F (5’-CTTGGTCATTTAGAGGAAGTAA-3’) and ITS2R (5’-MHTMHGTTCTTCATCGATMH-3’). The PCR reaction, amplification process, and purification steps followed the methodology outlined by Yang et al. (2018).

High-throughput sequencing was conducted on an Illumina HiSeq 2500 sequencer at Biomarker Tech. Initially, the raw data underwent paired-end (PE) read splicing using Flash v1.2.7. Subsequently, tags were filtered to obtain high-quality data, a process achieved with Trimmomatic v0.33. Chimeric sequences were eliminated using UCHIME v4.2 software to retain valid data. Tags were then clustered at a 97% similarity level using UCLUST within QIIME software. Operational taxonomic units (OTUs) were assigned for taxonomic annotation by referencing the Silvs and UNITE taxonomic databases. All raw sequencing data from each sample were deposited in the NCBI Sequence Read Archive (SRA) database under the accession number PRJNA719590.

### 2.3 Determination of rhizosphere soil allelochemicals

Based on reported allelopathic substances (Pervaiz et al., 2020; Chen Y. et al., 2022) and preliminary qualitative test reports, 14 substances with allelopathic potential were selected for quantitative testing. These substances include benzoic acid (BA), vanillin (VA), myristic acid (MA), methyl hexadecanoate (MH), scopoletin (SC), palmitic acid (PA), heptadecanoic acid (HA), linoleic acid (LA), linolenic acid (LIA), stearic acid (SA), arachidic acid (AA), 2,2′-methylenebis (6-tert-butyl-4-methylphenol) (AME), dioctyl phthalate (DOP), and erucylamide (EA).In the soil sample preparation process, a total of 5.00 g of freshly collected soil samples were introduced into a porcelain-evaporating dish, which included dried diatomaceous earth. Subsequently, these components were meticulously ground in a mortar until a homogeneous consistency akin to quicksand was achieved. The processed soil samples were then carefully transferred to an extraction tank, and 20.0 mL of an extraction solution (composed of n-hexane and acetone in a 1:1 ratio) was added. Employing an ASE, the sample was subjected to two pressurized extraction cycles, each entailing agitation at room temperature for 40 min. The resulting extract solution was meticulously decanted into a concentration bottle. The bottle underwent a sequence of three washes, each employing 3 mL n-hexane, and the washings were subsequently reintroduced into the concentration bottle. Subsequently, the extract solution was concentrated using a rotary evaporator, yielding a final volume within the range of 3–5 mL at an operating temperature of 35°C. In a further step, 20 mL of n-hexane was reintroduced and concentrated to a volume of 3 mL. This process was repeated twice to obtain a concentrated solution with a final volume of 3 mL. The eluate, purified through these procedures, was meticulously collected in a nitrogen-blow tube. Upon reaching a volume of 0.5 mL, ethyl acetate was added to adjust the final volume to 1 mL. The prepared samples were subsequently used for machine-based detection.

For gas chromatography, the inlet temperature was set at 220°C in splitless mode. Helium was employed as the carrier gas at a column flow rate of 1.16 mL/min. The temperature program involved a gradual increase at a rate of 10°C/min until it reached 220°C and was held at this temperature for 2 min. For mass spectrometry, the interface temperature was maintained at 280°C. The ion source temperature was set at 230°C. Data acquisition was performed using ion-selective scanning. The chromatographic column used for this analysis was an Rtx-5MS.

### 2.4 Fluorescent quantitative PCR of *Phytophthora parasitica* var. *nicotianae* in rhizosphere soil

The total soil DNA was utilized as a template for analysis. The target genes were amplified using specific primers: PF (5’-CGAAMHCAACCATACCACGAA-3’) and PR (5’-ATGAAGAACMHTMHGAACTMH-3’). The fluorescent quantitative PCR reaction was conducted in 30 μL reaction mixtures, consisting of 15 μL of qPCR Mix, 0.5 μL of Mg^2+^, 0.5μL of PF, 0.5μL of PR, 2 μL of DNA template, and ddH_2_O to reach a final volume of 30 μL. PCR thermal cycling conditions included an initial denaturation at 95°C for 3 min, followed by 35 cycles of denaturation at 95°C for 15 s, annealing at 65°C for 20 s, and extension at 72°C for 20 s. To quantify the black shank pathogen content in the soil, we constructed a standard curve using the tobacco black shank gene.

### 2.5 Tobacco agronomic traits, disease investigation, and analysis

For each treatment, we established five sampling points, each consisting of 50 representative tobacco plants. These sampling points were maintained from the time of tobacco transplantation until the harvest phase. Tobacco black shank disease was the main soil borne disease in the region and the word and it outbreaked at high temperature and high humidity condition(Mid-to-late July in Baishui). So a total of 250 plants from each treatment were investigate according to Ao et al. (2022). We conducted surveys every 5 days throughout July 2020 and subsequently calculated the average incidence rate according to the formula. We assessed the agronomic traits of the tobacco fields and determined the maximum leaf area index using the methodology described by Tang et al. (2020). After harvesting, we weighed the flue-cured tobacco leaves in each treatment and calculated the yield.


$$\text{Disease incidence rate}\left(\%\right)=\frac{\text{number of susceptible plants}}{\text{total number of investigated plants}}\times 100$$

### 2.6 Data processing and analysis

Origin 2022 was used to conduct variance analysis, generate heat maps, create correlation mappings, perform RDA analysis, and construct stacked column charts. To construct the interaction network, we utilized the R programming language, and for fitting curve estimation models, we used SPSS26.

## 3 Results

### 3.1 Characteristics of tobacco continuous cropping obstacles in different continuous cropping year

#### 3.1.1 Variations in growth and yield of tobacco across different continuous cropping years

**Figure 1:**
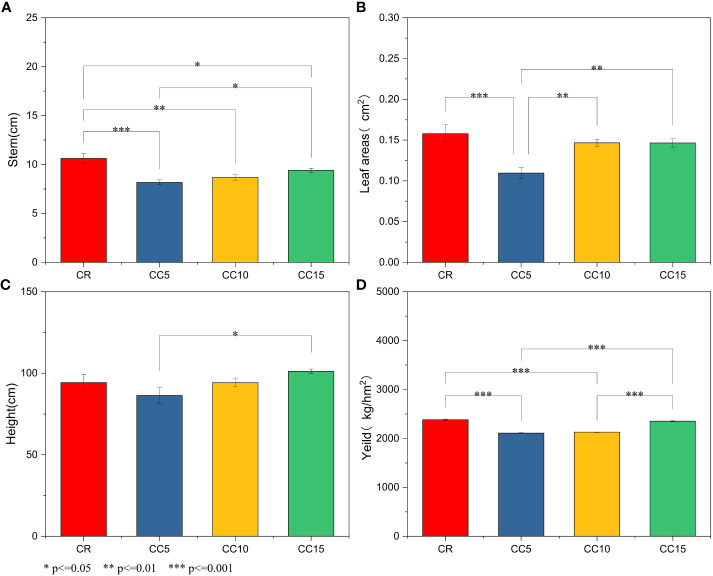
Agronomic traits and tobacco yields under different conditions. **(A)** Stem. **(B)** Leaf area. **(C)** Plant height. **(D)** Yield per ha. *, significant difference (*P* < 0.05). **/***, extremely significant difference (*P* < 0.01).

Tobacco stem circumference in all three continuous cropping treatments was significantly or extremely significantly lower than that in CR (*P<* 0.05 or *P<* 0.01), and the stem circumference of CC5 was the lowest among the three continuous cropping treatments, exhibiting a significant difference when compared to CC15 (**Figure 1A**). Additionally, maximum leaf area coefficient, and plant height in CC5 were the lowest and were significantly lower than those in CC15. No significant differences in stem circumference, maximum leaf area coefficient, and plant height were observed between CC10 and CC15 (**Figures 1B, C**). Significant variations were noted in tobacco yield across different continuous cropping years. CR and CC15 exhibited the highest yields, followed by CC10, while CC5 showed the lowest tobacco yield (**Figure 1D**). These findings highlight the substantial differences in tobacco growth under various continuous cropping durations. Growth was notably stunted after 5 years of continuous cropping, but with the extension of continuous cropping years, tobacco growth gradually normalized. After 15 years of continuous cropping, both the growth and yield of tobacco were not significantly different from those of tobacco cultivated for just 1 year.

#### 3.1.2 Incidence of tobacco black shank and pathogen accumulation across continuous cropping years

**Figure 2:**
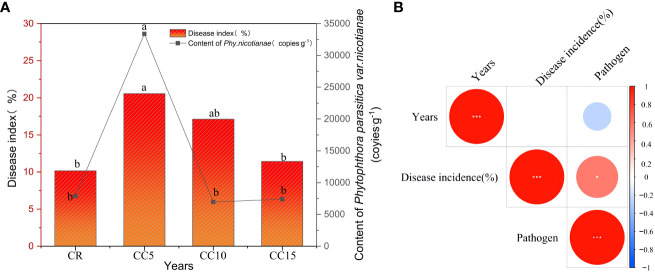
Tobacco disease index, pathogen accumulation, and correlation over continuous cropping years. **(A)** Tobacco disease index and pathogen accumulation. **(B)** Correlation among disease index, pathogen accumulation, and continuous cropping years. Different letters indicate significant differences according to the LSD test (P < 0.05). *significant correlation (*P* < 0.05). ***, extremely significant correlation (*P* < 0.01).

Tobacco black shank, a predominant soil-borne disease in the region, exhibited a higher incidence in the three continuous cropping treatments than in the CR treatment. Notably, CC5 treatment had a significantly higher incidence than CR (**Figure 2A**). Upon comparing the incidence of tobacco black shank within the three continuous cropping treatments, it was observed that CC5 had the highest incidence, which was not significantly different from CC10, but significantly higher than CC15. This pattern of incidence correlated with the accumulation of pathogens, with the highest accumulation of black shank pathogens occurring in CC5, significantly surpassing CC10, CC15, and CR. However, no significant difference was observed between CC10 and CC15. Correlation analysis indicated that there was no significant correlation between the incidence of tobacco black shank and continuous cropping years. Conversely, a negative correlation was observed between the accumulation of black shank pathogens and continuous cropping years. Peak accumulation was noted in CC5, after which pathogen enrichment slowed with the extension of continuous cropping years, gradually leading to a decline in its content (**Figure 2B**).

#### 3.1.3 Changes in the abundance of antagonistic bacterial flora against tobacco black shank pathogen across continuous cropping years

**Figure 3:**
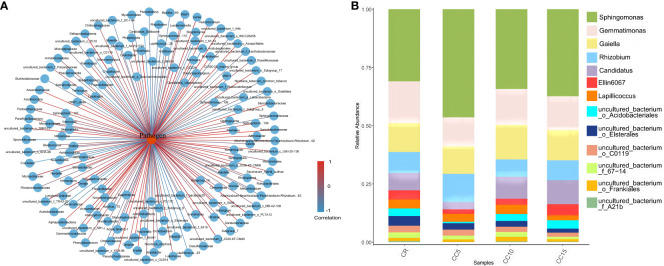
Bacteria interactions with *Phytophthora parasitica* var. *nicotianae* and the relative abundance of antagonistic bacteria. **(A)** Interaction network between *Phytophthora parasitica* var. *nicotianae* and bacteria at the genus level (> 0.1%). **(B)** Relative abundance of antagonistic bacterial flora in different continuous cropping years.

We constructed an interaction network to identify antagonistic bacterial flora against *Phytophthora parasitica* var. *nicotianae*, focusing on bacterial genera with a relative abundance exceeding 0.1%. Among the 65 bacterial species present in tobacco rhizosphere soil, we found a negative correlation with *Phytophthora parasitica* var. *nicotianae*, which indicated an antagonistic effect. Specifically, 13 bacterial species displayed strong antagonistic relationships with *Phytophthora parasitica* var. *nicotianae* (*P*< 0.05) (**Figure 3A**). We further analyzed the distribution of these 13 antagonistic bacterial groups across different continuous cropping years (**Figure 3B**). *Sphingomonas*, *Gemmatimonas*, *Gaiellales*, and *Rhizobium* were the dominant flora across all four treatments, collectively accounting for more than 60%. Notably, *Sphingomonas, Gemmatimonas*, and *Gaiellales* exhibited significant differences among treatments. The relative abundance of *Gemmatimonas* and *Gaiellales* in CR was significantly higher than that in CC5, with no significant difference observed compared to CC10 and CC15. For the different planting years, the relative abundance of *Gemmatimonas* and *Gaiellales* in CC10 and CC15 was notably higher. Except for *Lapillicoccus* and uncultured bacterium_o_C0119, the other seven bacterial groups showed significant differences across different continuous cropping years. The relative abundance of uncultured bacterium *Acidobacteriales, Candidatus*, uncultured bacterium_f_67-14, uncultured bacterium_o_*Elsterales*, uncultured bacterium_f*_A21b*, and *Ellin6067* was significantly lower than that in CC10 and CC15 with no significant difference between CC10 and CC15 (*P* > 0.05). These findings underscore the significant variations in the abundance of antagonistic bacteria within the tobacco rhizosphere across different continuous cropping treatments. In particular, the relative abundance of eight antagonistic bacteria in the CC5 treatment was significantly lower than that in CC10 and CC15. Conversely, the antagonistic bacterial flora in CC10 and CC15 exhibited a gradual increase, with no significant difference observed compared to CR.

### 3.2 Differential allelochemicals across continuous cropping years and their accumulation characteristics

**Figure 4:**
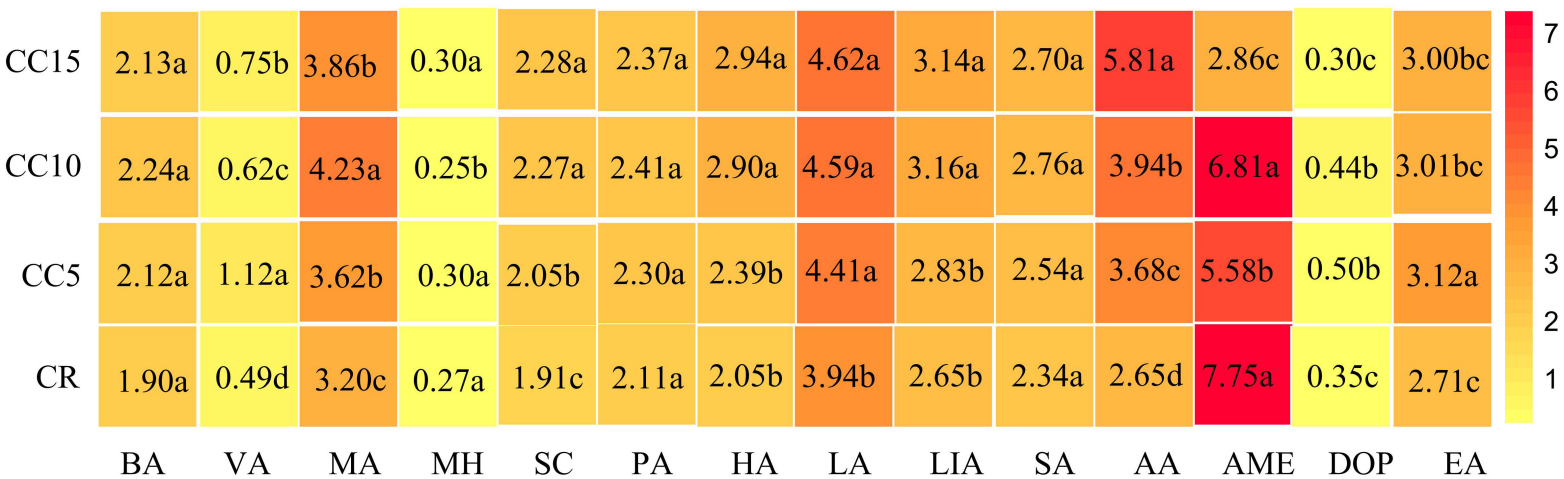
Heat map and differential analysis of 14 allelopathicals in tobacco rhizosphere. Different letters indicate significant differences according to the LSD test (*P*< 0.05). BA, benzoic acid; VA, vanillin; MA, myristic acid; MH, methyl hexadecanoate; SC, scopoletin; PA, palmitic acid; HA, heptadecanoic acid; LA, linoleic acid; LIA, linolenic acid; SA, stearic acid; AA, arachidic acid; AME, 2,2′-methylenebis (6-tert-butyl-4-methylphenol); DOP, dioctyl phthalate; EA, erucylamide.

We detected 14 allelopathicals using GC-MS for all four treatments (**Figure 4**). Variance analysis revealed significant differences in the 10 allelopathicals between CR and various continuous cropping years, except for BA, PA, SA, and LA (*P<* 0.05). Subsequently, we analyzed the allelopathicals across different continuous cropping years. The contents of VA and EA were notably the highest in the CC5 treatment, significantly surpassing that of CC10 and CC15. Moreover, CC15 exhibited significantly higher levels of VA and EA than CC10 did. In contrast, MA, AME, and DOP were most abundant in the CC10 treatment, with significantly higher levels than in the CC5 and CC15 treatments. Additionally, SC, HA, LIA, and MH were significantly more abundant in CC15 than in CC5 or CC10. These findings indicate that all 14 allelochemicals were detectable across the different continuous cropping treatments. Among them, 10 allelochemicals displayed significant differences in three continuous tobacco rhizosphere treatments. Furthermore, these 10 allelochemicals exhibited diverse accumulation trends in various continuous tobacco rhizosphere environments.

We established linear functions for the curve estimation model with the dependent variable (y) representing the 10 allelopathicals that exhibited significant differences between CR and various continuous cropping years. The independent variable (t) represents the tobacco planting years, and this analysis allowed us to examine the accumulation characteristics of these 10 differential allelopathicals. The accumulation trends of VA and EA followed a cubic polynomial curve model (*R^2^* = 0.978 and *R^2^* = 0.807). In contrast, the accumulation trends of MA, MH, and DOP conformed to a quadratic polynomial growth model (*R^2^* = 0836, *R^2^* = 0.728, and *R^2^* = 0.935). Additionally, the accumulation trends of SC, HA, LIA, AA, and AME followed a general growth model (**Table 1**). Based on the growth patterns observed in tobacco and the occurrence of diseases, we speculated that VA, MA, MH, DOP, and EA may be related to the continuous cropping obstacles observed in different continuous cropping years.

**Table 1:** Curve estimation models for 10 differential allelopathicals.

Allelopathicals	Fitting Model	Decisive factor	Significance	regression equation
		R2	P	
VA	cubic polynomial curve	0.978	0.000	y=-0.015+0.043t^3^-0.010t^2^+0.001t
MA	quadratic polynomial growth	0.836	0.000	y=2.467+0.590t^2^-0.045t
MH	quadratic polynomial growth	0.728	0.003	y=0.301-0.027t^2^+0.003t
SC	growth model	0.599	0.003	y=EXP(0.647+0.021t)
HA	growth model	0.612	0.003	y=EXP(0.733+0.041t)
LIA	growth model	0.593	0.003	y=EXP(0.975+0.020t)
AA	growth model	0.820	0.000	y=EXP(1.039+0.079t)
AME	growth model	0.737	0.000	y=EXP(2.167-0.100t)
DOP	quadratic polynomial growth	0.935	0.000	y=0.255+0.082t^2^-0.008t
EA	cubic polynomial curve	0.807	0.003	y=-1.059+1.938t^3^-0.449t^2^+0.027t

### 3.3 Screening key allelopathicals affecting tobacco continuous cropping obstacles

#### 3.3.1 Impact of differential allelopathicals in tobacco rhizosphere on tobacco growth

**Figure 5:**
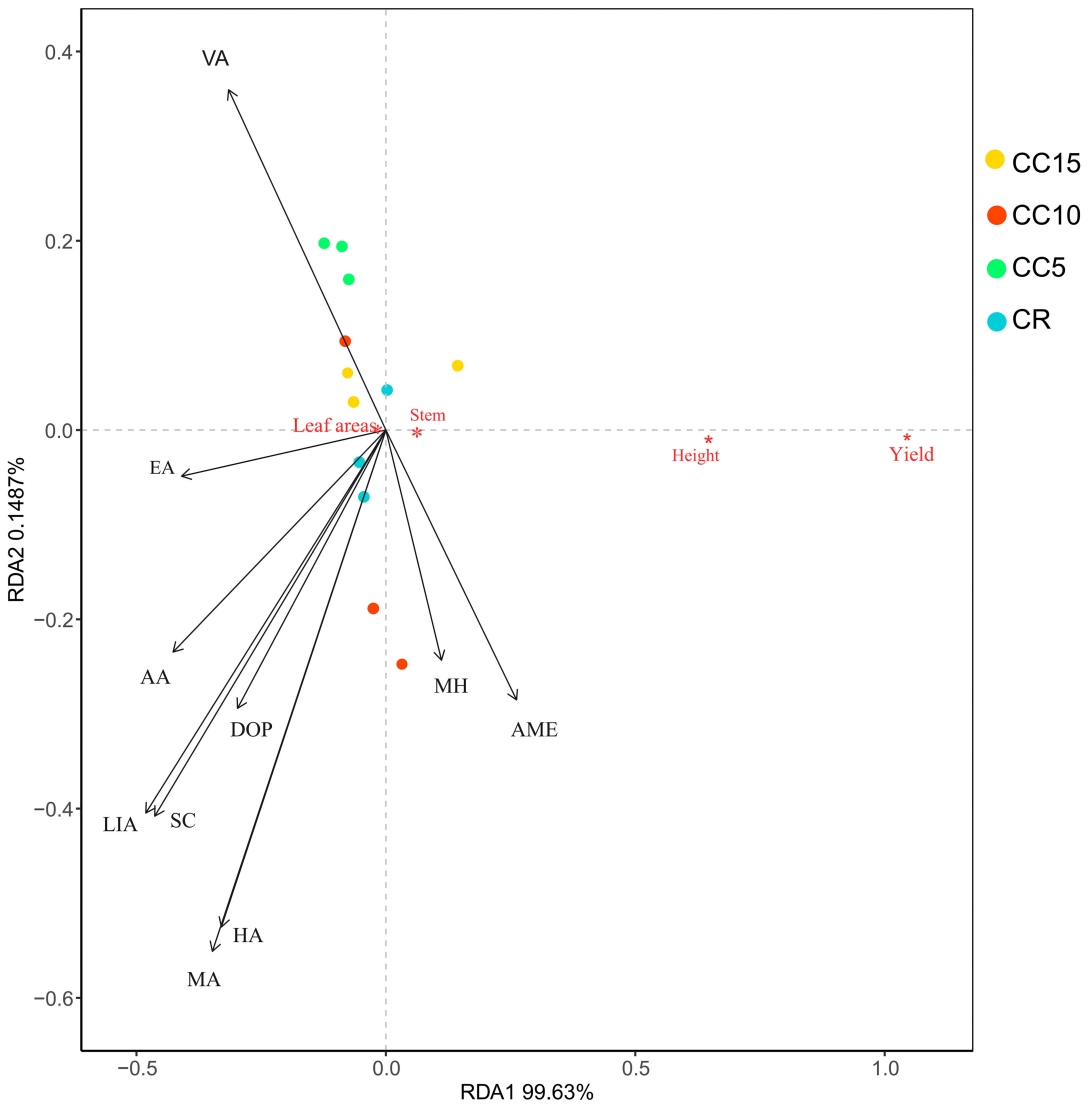
RDA analysis of differential allelopathicals and tobacco growth.

The RDA analysis revealed that tobacco growth in CR and CC15 was similar, whereas CC5 and CC10 belonged to a distinct group (**Figure 5**). When considering the effects of the 10 differential allelochemicals on tobacco growth, they ranked from strong to weak impact as follows: MA, HA, VA, LIA, SC, DOP, AA, MH, and EA. Except for MH and AME, the other eight allelochemicals displayed negative correlations with stem circumference, tobacco plant height, and yield. Among these, MA, HA, and VA had the most pronounced negative effects, indicating that MA, HA, and VA significantly contributed to the observed effects on tobacco growth.

#### 3.3.2 Interaction network among differential allelochemicals, *Phytophthora parasitica* var. *nicotianae*, and antagonistic microbial communities

**Figure 6:**
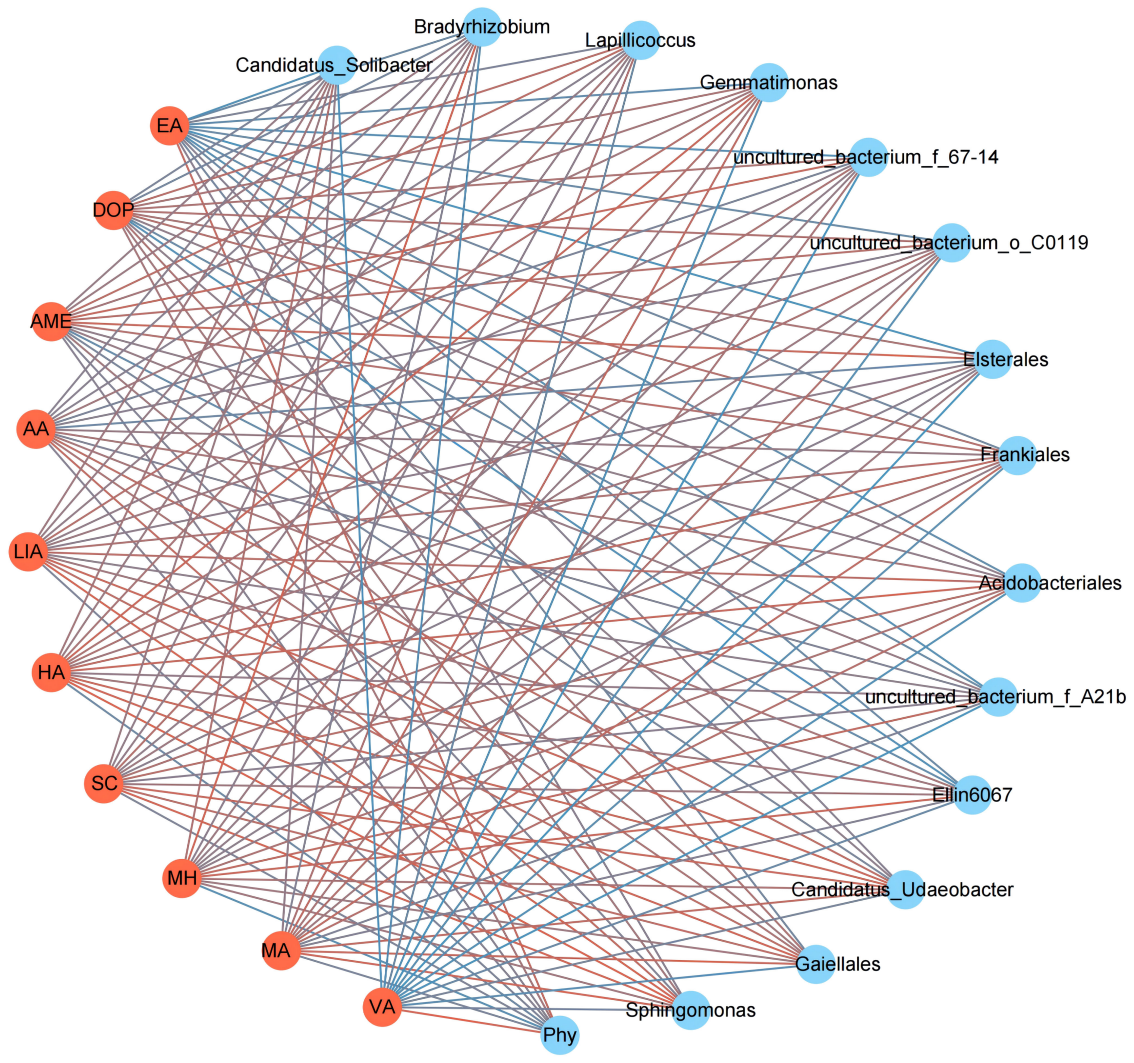
Interaction network between 10 differentiated allelopathicals, *Phytophthora parasitica* var. *nicotianae* and antagonistic bacteria. Phy, *Phytophthora parasitica* var. *nicotianae;* Red line, positive correlation; Blue line, negatively correlation (*P* < 0.05).

The interaction network analysis revealed specific correlations between allelopathicals and *Phytophthora parasitica* var. *nicotianae* as well as antagonistic microbial flora (**Figure 6**). VA and EA exhibited positive correlations with *Phytophthora parasitica* var. *Nicotianae* and negative correlations with 13 antagonistic microbial flora (*P<* 0.05). DOP was positively correlated with *Phytophthora parasitica* var. *nicotianae* and negatively correlated with Candidatus, Bradyrhizobium, uncultured_bacteria_f_A21b, and Ellin6067. MA, MH, SC, HA, and LIA were positively correlated with most antagonistic bacteria and negatively correlated with *Phytophthora parasitica* var. *nicotianae.*

### 3.4 Mechanisms of key allelochemicals in causing tobacco continuous cropping obstacles

**Figure 7:**
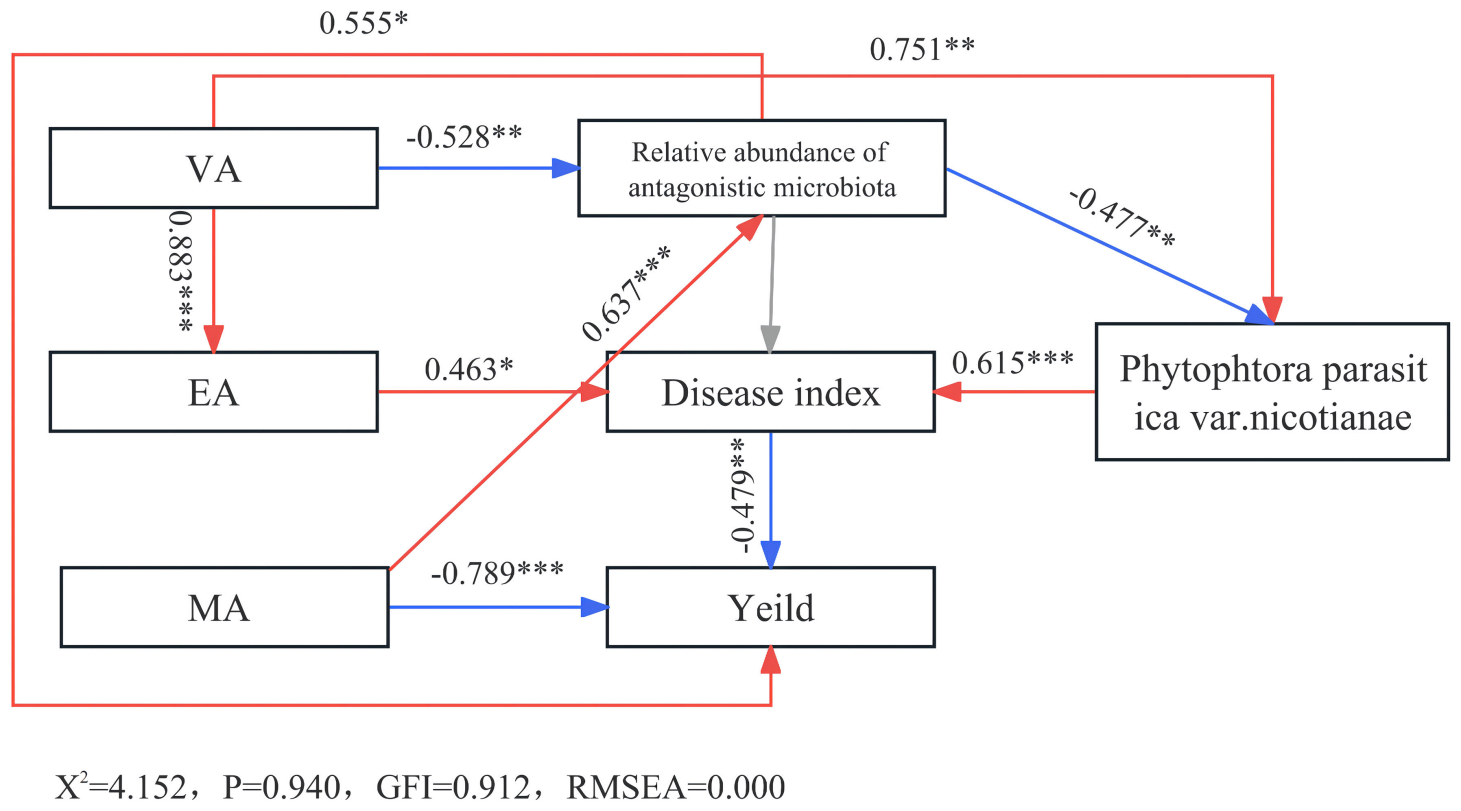
Structural equation model of main allelopathicals in tobacco rhizosphere with tobacco disease and yield. *significant correlation (*P* < 0.05). **/***, extremely significant correlation (*P* < 0.01).

Based on the above analysis, MA, VA, and EA were found to be significant allelochemicals. The structural equation model revealed a positive correlation between VA and *Phytophthora parasitica* var. *nicotianae*, directly enhancing its accumulation (**Figure 7**). Conversely, it hindered the growth of beneficial bacteria, consequently promoting the accumulation of *Phytophthora parasitica* var. *nicotianae* and fostering soil-borne diseases. Furthermore, VA stimulated the accumulation of EA, exacerbating diseases and reducing crop yields. MA, on the other hand, directly impeded tobacco growth, resulting in persistent cropping obstacles. EA contributed to yield reduction by intensifying disease occurrence. These findings underscore the direct and indirect roles of these key allelopathicals in causing obstacles to continuous tobacco cropping. Notably, VA’s indirect influence surpassed its direct impact, as it encouraged pathogen proliferation, impeded beneficial bacterial enrichment, and collaborated with EA to compose tobacco continuous cropping obstacles. With its multifaceted action, VA appears to be the most influential allelopathic factor contributing to the persistence of these cropping obstacles.

## 4 Discussion

Continuous monoculture leads to the accumulation of soil-borne pathogens, a decline in beneficial bacteria, and disruption of the microbial community, which is often referred to as the plant’s second genome. This results in poor crop growth, soil-borne disease outbreaks, and significant economic loss. This phenomenon is widespread across various crops, including food crops (Yao et al., 2020), cash crops (Gao et al., 2019; Xi et al., 2019), vegetables (Gao et al., 2019), and medicinal materials (Deng et al., 2023). In our study, we observed that tobacco growth was severely hindered after 5 years of continuous cropping, marked by a high incidence of tobacco black shank and pathogen accumulation, indicating noticeable obstacles. After 15 years of continuous cropping, we observed an improvement in tobacco growth and a reduction in disease occurrence, in contrast to the findings of Chen et al. (2018), who reported obstacles to continuous cropping 25 years after tobacco planting. This discrepancy may be attributed to the influence of the different varieties. Previous studies have demonstrated significant variations in soil microbial structure among different genotypes of the same crop (Weinert et al., 2011), influencing responses to continuous cropping by affecting soil nutrient content and crop disease resistance (Priyanka et al., 2020; Maria-Soledad et al., 2021). For example, studies on soybean varieties have revealed that tolerant varieties exhibit higher levels of beneficial bacteria, increased available nutrient content, and enhanced activities of nitrogen and phosphorus cycling enzymes than sensitive varieties (Liu et al., 2023). Microbial functions may serve as the primary mechanisms explaining these phenomena. Long-term continuous cropping in the wheat rhizosphere enriches antagonistic *Pseudomonas fluorescens* and triggers the secretion of antifungal compounds, mitigating the challenges associated with continuous cropping (Berendsen et al., 2012). In this study, we observed that the relative abundance of nine antagonistic bacterial groups in the 15-year continuous cropping rhizosphere was higher than in the 5-year continuous cropping treatment. There was no significant difference in the relative abundance of antagonistic bacterial genera in the tobacco rhizosphere after 1 and 10 years of continuous cropping. This may explain the increasing severity of tobacco continuous cropping obstacles after 5 years but their alleviation after 15 years. Analysis of antagonistic bacterial groups revealed relatively abundant genera, such as *Gemmatimonas, Gaiellales, Candidatus*, and *Ellin6067. Gemmatimonas* promotes plant growth and pathogen resistance (Xiong et al., 2015; Yang et al., 2018; Karthika et al., 2020). *Gaiellales* thrives in healthy plants, particularly in soils with high organic matter content (Lin et al., 2019; Pang et al., 2022). *Candidatus* is a crucial microbial community in wheat rhizospheres that positively influences crop yields and soil functions (Fan et al., 2021) and participates in soil pollutant transformations (Chen C. et al., 2022). *Ellin6067* aids in pollutant degradation and enhances plant growth (Yang et al., 2021). These results suggest that long-term continuous cropping promotes resilience of beneficial bacteria to adverse conditions, enriching bacterial groups related to plant growth, pathogen resistance, and pollutant degradation. This enrichment contributes to the formation of disease-suppressing soils and helps mitigate continuous cropping obstacles. However, the underlying reasons for the changes in tobacco rhizosphere microbial community structure with varying continuous cropping years remain unclear. Further exploration is needed to deepen our understanding of the mechanisms underlying obstacles to tobacco continuous cropping.

A study by Rial et al. (Rial et al., 2014) demonstrated that long-term continuous cropping plants release substances known as autotoxic allelopathicals into their rhizosphere, inhibiting their own growth. As continuous cropping years increase, these autotoxic allelopathicals accumulate along with soil acidification, hindering the growth and reproduction of beneficial microorganisms (Wu et al., 2015). These allelopathicals are likely the root cause of changes in rhizosphere microorganisms of continuously cropped plants, ultimately affecting plant growth. We identified 14 allelopathicals using GC-MS, of which 10 exhibited significant differences across various continuous cropping years. Further analysis revealed that the levels of VA and EA were highest after 5 years of continuous cropping, followed by a downward trend as continuous cropping years extended, conforming to a cubic polynomial growth pattern. Conversely, the levels of MA, AME, and DOP were the highest after 10 years of continuous cropping, with the accumulation of MA and DOP following a quadratic polynomial growth pattern. Allelochemicals adhering to quadratic or cubic polynomial growth patterns appeared to correlate more closely with tobacco growth and disease occurrence trends, potentially playing a role in tobacco continuous cropping obstacles. The results of the RDA indicated that MA, HA, and VA were the top three allelopathicals that significantly inhibited tobacco growth. Chen et al. (2011) verified that elevated VA concentration (1–4 nmol/L) notably inhibits eggplant seedling growth and increases the incidence of verticillium wilt disease. Myristic acids contribute to the exacerbation of tobacco bacterial wilt by facilitating the colonization of pathogenic bacteria, consequently affecting tobacco growth (Li et al., 2016). Over short-term continuous cropping years, HA exhibits an enrichment trend correlated with an increase in tobacco continuous cropping years, potentially linked to the occurrence of continuous cropping obstacles (Pan et al., 2023). However, the current evidence lacks direct confirmation of the autotoxicity of this substance, necessitating further investigation. Network analysis further revealed that VA and EA could stimulate the accumulation of *Phytophthora parasitica* var. *nicotianae* while inhibiting the enrichment of 13 antagonistic bacteria. This may be attributed to allelopathicals serving as carbon sources that boost the proliferation of pathogenic microorganisms (Carrara et al., 2018), enabling them to dominate ecological niches, suppress beneficial bacteria growth, trigger disease outbreaks, and contribute to continuous cropping obstacles (Furtak and Gajda, 2018). In light of these findings, it is evident that MA, VA, and EA are likely key allelopathicals responsible for disrupting the rhizosphere microbial community structure, impeding tobacco growth, and leading to tobacco black shank disease.

Wu et al. (Wu, 2010) isolated several allelopathicals, including VA, from tobacco rhizosphere. Bioassays indicated that vanillin had a weaker inhibitory effect on tobacco seedlings than benzoic acid or p-hydroxybenzoic acid. Our findings suggest that the direct inhibitory effect of vanillin on tobacco growth was less pronounced than that of MA. This implies that different allelopathicals affect tobacco differently, underscoring the importance of identifying key allelopathicals and elucidating their mechanisms of action for more in-depth analysis of their pathways. Structural equation model results revealed that MA and EA primarily induced continuous cropping obstacles through direct toxicity to tobacco. VA featured prominently in the model due to its multifaceted actions. On one hand, it promoted the accumulation of *Phytophthora parasitica* var. *nicotianae*, inhibited beneficial bacterial enrichment, disrupted the tobacco rhizosphere microbial community, and exacerbated obstacles to continuous cropping. For example, low concentrations of VA encouraged eggplant Fusarium wilt, whereas high concentrations enhanced eggplant autotoxicity and inhibited eggplant growth (Chen et al., 2011). On the other hand, VA can stimulate the accumulation of EA, intensifying its direct inhibitory effect on tobacco. Wu et al. (Wu et al., 2016) confirmed that a mixture of syringic acid and phenolic acid exhibited a stronger allelopathic effect on *Radix pseudostellariae* than a single allelopathic effect, highlighting the interactions between substances that affect continuous cropping obstacles. In summary, our results indicate that MA and EA primarily impact tobacco growth through direct toxicity, whereas VA disrupts the microbial community structure and promotes EA accumulation, jointly exacerbating continuous cropping obstacles. Regardless of the scenario, key allelopathicals consistently play a pivotal role in the crop rhizosphere, serving as crucial links between rhizosphere allelopathicals, microorganisms, and crop growth, ultimately influencing the occurrence of continuous cropping obstacles. Based on our findings, we speculated that VA may be a key allelopathical in the tobacco rhizosphere, and its changing content over continuous cropping years could serve as an essential internal mechanism to alleviate obstacles in 15 years of continuous cropping.

## 5 Conclusion

The most pronounced challenges were observed in the CC5 category, with yield reduction, tobacco black shank outbreaks, and diminished beneficial flora. In contrast, CC15 exhibited a substantial reduction in challenges as the continuous cropping persisted. This was marked by tobacco growth recovery, a reduction in disease incidence, and an increased abundance of beneficial bacteria. This phenomenon was closely mirrored by allelochemical accumulation patterns in the tobacco rhizosphere. Notably, among these allelochemicals, VA exhibited significant variation in the tobacco rhizosphere across different planting years, following a cubic polynomial curve model. Although its direct inhibitory effect on tobacco was weaker than that of MA, VA played a pivotal role in fostering pathogen proliferation, inhibiting the enrichment of beneficial bacteria, and synergistically exacerbating the challenges associated with continuous cropping, particularly with EA. This mechanism is essential for understanding the occurrence and alleviation of continuous cropping obstacles in tobacco, particularly 15 years after planting.

## Data availability statement

The datasets presented in this study can be found in online repositories. The names of the repository/repositories and accession number(s) can be found below: NCBI Sequence Read Archive (SRA) database under the accession number PRJNA719590.

## Author contributions

FZ: Conceptualization, Data curation, Formal analysis, Investigation, Methodology, Resources, Validation, Visualization, Writing – original draft, Writing – review & editing. YP: Formal analysis, Investigation, Methodology, Resources, Writing – review & editing, Data curation. XZ: Formal analysis, Resources, Validation, Writing – review & editing. GD: Methodology, Resources, Validation, Writing – review & editing. XL: Data curation, Formal analysis, Writing – review & editing. YX: Data curation, Formal analysis, Writing – review & editing. LT: Conceptualization, Funding acquisition, Project administration, Resources, Supervision, Validation, Writing – review & editing.
